# Exogenous Asymmetric Dimethylarginine (ADMA) in Pathogenesis of Ischemia-Reperfusion-Induced Gastric Lesions: Interaction with Protective Nitric Oxide (NO) and Calcitonin Gene-Related Peptide (CGRP)

**DOI:** 10.3390/ijms15034946

**Published:** 2014-03-20

**Authors:** Marcin Magierowski, Katarzyna Jasnos, Zbigniew Sliwowski, Marcin Surmiak, Gracjana Krzysiek-Maczka, Agata Ptak-Belowska, Slawomir Kwiecien, Tomasz Brzozowski

**Affiliations:** Department of Physiology, Jagiellonian University Medical College, Grzegorzecka Street 16, Cracow 31-531, Poland; E-Mails: m.magierowski@uj.edu.pl (M.M.); k.jasnos@interia.pl (K.J.); agazs@poczta.fm (Z.S.); marcin.surmiak@uj.edu.pl (M.S.); gracjana98@gmail.com (G.K.-M.); agata.ptak-belowska@uj.edu.pl (A.P.-B.); skwiecien@cm-uj.krakow.pl (S.K.)

**Keywords:** asymmetric dimethyl arginine, gastric mucosa, ischemia/reperfusion, farnesoid X receptor, gastric damage, sensory neurons, calcitonin gene-related peptide, capsaicin

## Abstract

Asymmetric dimethylarginine (ADMA) is an endogenous nitric oxide (NO) synthesis inhibitor and pro-inflammatory factor. We investigated the role of ADMA in rat gastric mucosa compromised through 30 min of gastric ischemia (I) and 3 h of reperfusion (R). These I/R animals were pretreated with ADMA with or without the combination of l-arginine, calcitonin gene-related peptide (CGRP) or a small dose of capsaicin, all of which are known to afford protection against gastric lesions, or with a farnesoid X receptor (FXR) agonist, GW 4064, to increase the metabolism of ADMA. In the second series, ADMA was administered to capsaicin-denervated rats. The area of gastric damage was measured with planimetry, gastric blood flow (GBF) was determined by H_2_-gas clearance, and plasma ADMA and CGRP levels were determined using ELISA and RIA. ADMA significantly increased I/R-induced gastric injury while significantly decreasing GBF, the luminal NO content, and the plasma level of CGRP. This effect of ADMA was significantly attenuated by pretreatment with CGRP, l-arginine, capsaicin, or a PGE_2_ analogue. In GW4064 pretreated animals, the I/R injury was significantly reduced and this effect was abolished by co-treatment with ADMA. I/R damage potentiated by ADMA was exacerbated in capsaicin-denervated animals with a further reduction of CGRP. Plasma levels of IL-10 were significantly decreased while malonylodialdehyde (MDA) and plasma TNF-α contents were significantly increased by ADMA. In conclusion, ADMA aggravates I/R-induced gastric lesions due to a decrease of GBF, which is mediated by a fall in NO and CGRP release, and the enhancement of lipid peroxidation and its pro-inflammatory properties.

## 1. Introduction

Under physiological conditions free l-arginine (l-arg) can be metabolized by arginases to l-ornithine and urea, or it can serve as a substrate for nitric oxide synthase (NOS) to synthesize nitric oxide (NO) and l-citrulline [1] However, the protein-incorporated l-arg residues are methylated through the action of enzymes called protein arginine methyltransferases (PRMTs) known to generate N^G^-monomethyl-l-arginine (l-NMMA), asymmetric dimethyl arginine (ADMA) or symmetric dimethyl arginine (SDMA). ADMA and SDMA are present in human plasma at a concentration of 500 nM–1 μM, while l-NMMA concentration is about 10 times lower [2,3]. Only asymmetrically methylated forms of l-N^G^-monomethyl arginine (l-NMMA) and ADMA, but not SDMA, exert an inhibitory effect on NOS [4]. Both l-NMMA and ADMA are nonspecific NO inhibitors and compete with l-arg for each of the three isoforms of NOS, endothelial nitric oxide synthase (eNOS), neural nitric oxide synthase (nNOS), and inducible nitric oxide synthase (iNOS) [5]. Endogenous methylarginine can inhibit 10% of NOS activity. Under a variety of pathological conditions such as cardiovascular disorders, plasma ADMA level is highly elevated from three- up to nine-fold, which corresponds to 30%–70% inhibition of NO production from eNOS [6,7]. Recently, we reported that the concentration of ADMA is increased in animals exposed to stress and ischemia/reperfusion I/R [8] but little is known about the factors that can afford gastroprotection against the damage increased by ADMA in a stomach compromised by I/R. In particular, whether potent gastroprotectants such as capsaicin applied in a small “protective” dose or exogenous calcitonin gene related peptide (CGRP), the potent vasodilatatory peptide released from sensory afferent nerves, could affect an increase in plasma ADMA levels in rats with acute gastric mucosal I/R injury have not been so far examined.

Apart from modulation of NO production, ADMA is also involved in organ inflammatory reactions. Previous studies revealed that ADMA participates in the process of atherogenesis by enhancing production of proinflammatory cytokines such as tumor necrosis factor-alpha (TNF-α) [9], monocyte adhesion [10], and oxidized low-density lipoprotein accumulation in macrophages [11]. Additionally, ADMA was shown to induce cell apoptosis in a dose-dependent manner and contribute to oxidative processes [12,13]. Dimethylarginine dimethylaminohydrolase (DDAH) metabolizes ADMA (but not SDMA) to dimethylamine and citrulline. In rats, more than 90% of ADMA is eliminated due to the activity of DDAH1, which plays an essential role in maintaining NO bioavailability [14]. Interestingly, activation of the farnesoid X receptor (FXR) through synthetic FXR agonists, such as 3-(2,6-dichlorophenyl)-4-(3′-carboxy-2-chlorostilben-4-yl)-oxymethyl-5-isopropylisoxazole (GW4064), was shown to increase DDAH1 expression [15]. It was proposed that modulating DDAH1 activity through the FXR receptor agonists could be a therapeutic target for treating reduced NO bioavailability in congestive heart failure and other cardiovascular diseases [15]. In human hepatocytes, the activation of FXR with its agonist, GW4064, increased activity of DDAH-1 and decreased plasma circulating levels of ADMA, resulting in significant protection against cisplatin-induced toxicity [16]. Since these findings provided an insight into the mechanism by which FXR may affect NO levels through modulation of blood ADMA levels via direct regulation of hepatic DDAH1 expression and activity [17], we tested the hypothesis that GW4064 could afford protection of I/R-induced gastric lesions in the absence and presence of ADMA. Additionally, the determined effect of activation and suppression of sensory nerves releasing CGRP [18] in either protection against I/R injury or exacerbation of these lesions by ADMA have yet to be explored.

The excessive generation of reactive oxygen species (ROS), adhesion of neutrophils to endothelial cells, the activation of various proinflammatory mediators and the alternation in gastric acid secretion have been implicated in the pathogenesis of I/R-induced gastric mucosal injury [19]. Under pathological conditions, such as damage by I/R, the superoxide (O_2_^•−^) reacts with NO to produce peroxynitrite (ONOO^−^), one of the most powerful and highly damaging oxidants [20]. Interestingly, more than a 1000-fold production of NO and O_2_^•−^ increases the formation of ONOO^−^ by a 1,000,000-fold [21]. The protective effect of NO synthase inhibitors against I/R injury has been described in various tissues [22–24]. It is of interest that donors of NO have also been shown to reduce gastric I/R injury [25]. Recent evidence has revealed that ADMA, as a NO inhibitor, may contribute to the pathogenesis of acute gastric mucosa injury [26–28] but little is known about whether administration of ADMA can influence I/R-induced gastric lesions. In this study, we administered ADMA in gradual concentrations to determine the effect of this NO synthase inhibitor on gastric I/R lesions, plasma ADMA levels, gastric luminal NO concentration, and changes in the gastric blood flow (GBF). We also determined the effects of ADMA in rats with capsaicin-deactivated sensory neurons depleted of vasodilatory neuromediator CGRP. Finally, we examined the effect of a synthetic prostaglandin (PG) E_2_ analog, the prototype of gastroprotective substances [29,30], on I/R-induced gastric lesions and the accompanying changes in plasma anti-inflammatory (interleukin (IL)-10) and proinflammatory cytokine (TNF-α) levels with or without ADMA administration.

## 2. Results and Discussion

**Figure 1.**
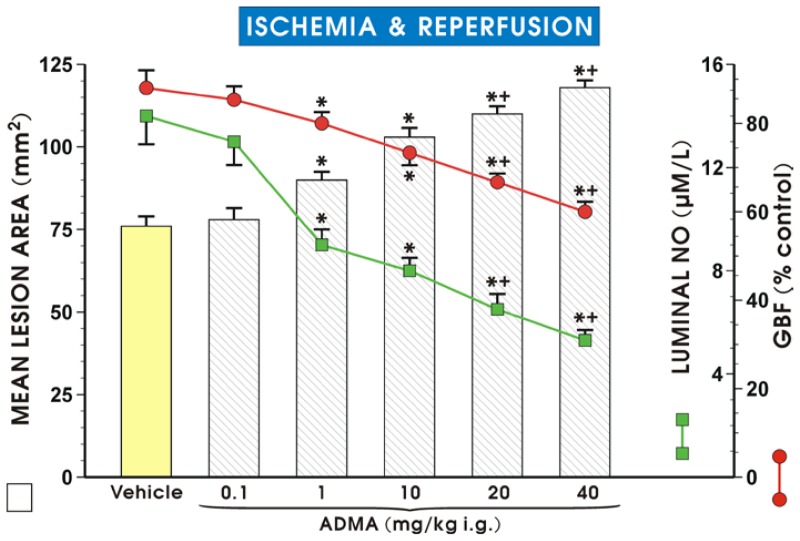
Mean lesion area, gastric blood flow (GBF) level, and gastric luminal NO concentration after application of vehicle (saline) or ADMA administered in graded doses (0.1–40 mg/kg i.g.) followed by exposure to ischemia/reperfusion. Results are mean ± S.E.M. of seven rats per group. Significant change (*p* < 0.05) as compared with the respective values in vehicle-control group is indicated by an asterisk. A cross and asterisk indicate significant change (*p* < 0.05) when compared to the values obtained in vehicle-controls and in those pretreated with ADMA (1 or 10 mg/kg i.g.).

Figure 1 shows that ADMA (0.1–40 mg/kg i.g.) pretreatment dose-dependently increased the mean area of I/R-induced gastric lesions as compared with the vehicle-control group, which was pretreated with vehicle (saline) (*p* < 0.05). ADMA administered at a dose of 0.1 mg/kg failed to affect the area of I/R injury but a significant increase in lesion area was observed starting from a dose of 1 mg/kg of ADMA, and this effect was potentiated by ADMA administered in higher doses up to 40 mg/kg. This exacerbating effect of ADMA on I/R injury was accompanied by a dose-dependent decrease in GBF (*p* < 0.05). The GBF was significantly decreased by pretreatment with ADMA applied in higher doses of 20 and 40 mg/kg (*p* < 0.05) as compared to that in animals pretreated with ADMA in doses of 1 and 10 mg/kg. The luminal NO concentration was significantly decreased by ADMA given in a dose of 1 or 10 mg/kg (*p* < 0.05) as compared with the vehicle-control group, and a further significant fall in luminal NO concentration was observed when ADMA was applied in higher doses of 20 and 40 mg/kg as compared with the respective values of lower doses of ADMA (*p* < 0.05) (Figure 1). Of note, our preliminary studies revealed that ADMA administered alone in a dose of 20 mg/kg (i.g.) did not induce gross damage detectable in the gastric mucosa and this effect has been omitted for the sake of clarity.

In intact animals, not exposed to I/R, the plasma ADMA levels were negligible and reached a value of 0.22 ± 0.03 μmol/L. As shown in Table 1, ADMA administered i.g. in graded doses ranging from 0.1 up to 40 mg/kg, produced a dose-dependent increase in the plasma level of ADMA. The increase in plasma ADMA levels achieved in rats treated with exogenous ADMA applied in a dose of 20 and 40 mg/kg reached significantly higher values (*p* < 0.05) than those recorded when ADMA was administered in smaller doses of 1 or 10 mg/kg.

Data presented in Table 2 indicates that pretreatment with ADMA significantly reduced the luminal NO concentration determined after ischemia but before the subsequent reperfusion period (*p* < 0.05), as compared to that measured in vehicle-pretreated controls before reperfusion. Likewise, the luminal concentration of NO was significantly inhibited in ADMA-treated animals at the end of 3.5 h of I/R (*p* < 0.05) as compared to vehicle-control animals (Table 2).

**Figure 2.**
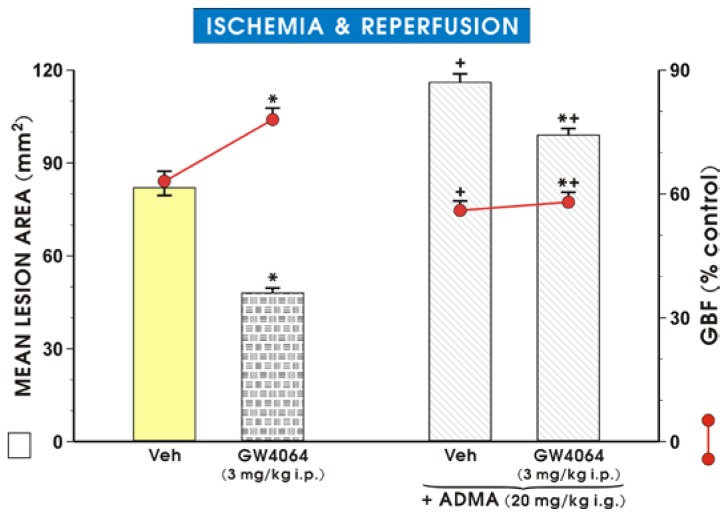
Mean lesion area and the changes in gastric blood flow (GBF) in vehicle and GW4064 (3 mg/kg i.p.) pretreated rats exposed to 3.5 h of ischemia/reperfusion in the absence and the presence of ADMA (20 mg/kg i.g.). Results are mean ± S.E.M of six rats per group. Significant change (*p* < 0.05) as compared with the respective values in vehicle-control group is indicated by an asterisk. A cross indicates a significant difference (*p* < 0.05) compared with the vehicle control treated group not treated with ADMA. A cross and an asterisk indicate a significant change (*p* < 0.05) compared to the values obtained in rats treated with GW4064 without ADMA.

Figure 2 shows that the FXR agonist, GW4064, which was reported to increase the DDAH1 expression and thereby decrease plasma circulating levels of ADMA [17], significantly reduced the I/R-induced gastric lesions. This effect was accompanied by a significant increase in the GBF as compared to respective values obtained in vehicle-control animals. Concurrent treatment with ADMA reversed the GW4064-induced decrease in the area of I/R-induced gastric lesions and the accompanying increase in the GBF caused by this FXR agonist.

**Figure 3.**
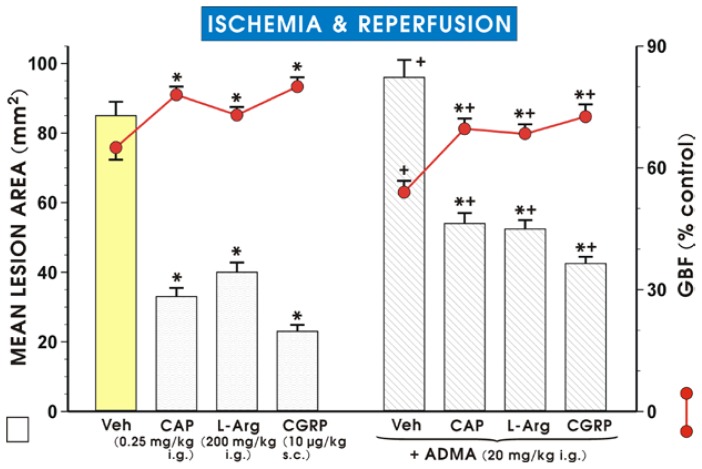
Mean lesion area and the changes in gastric blood flow (GBF) after application of a small “protective” dose of capsaicin (CAP, 0.25 mg/kg i.g.), l-arginine (l-Arg, 200 mg/kg i.g.) or CGRP (10 μg/kg s.c.). Each was applied alone and in combination with ADMA (20 mg/kg i.g.), followed by exposure to ischemia/reperfusion. Results are mean ± S.E.M of 6–8 rats per group. Significant change (*p* < 0.05) as compared with the respective values in the vehicle-control group, is indicated by an asterisk. A cross indicates a significant change (*p* < 0.05) compared to the values obtained in the vehicle-control group. An asterisk and a cross indicate a significant change (*p* < 0.05) as compared to the values obtained in rats treated with vehicle, capsaicin, l-arg, or CGRP without ADMA.

The area of I/R-induced gastric lesions was significantly decreased in rats pretreated with low dose of capsaicin (0.25 mg/kg i.g.) to stimulate sensory afferent nerves, and in those pretreated with l-arg (200 mg/kg i.g.) and CGRP (10 μg/kg s.c.), as compared with the vehicle (saline) pretreated group (*p* < 0.05) (Figure 3). The protective effects of capsaicin, l-arg and CGRP were accompanied by a significant increase in GBF (*p* < 0.05). Co-administration of ADMA (20 mg/kg i.g.) with capsaicin, l-arg or CGRP significantly reduced the protective effect of capsaicin, l-arg and CGRP administered alone, and these effects were accompanied by a significant decrease in the GBF (Figure 3).

**Figure 4.**
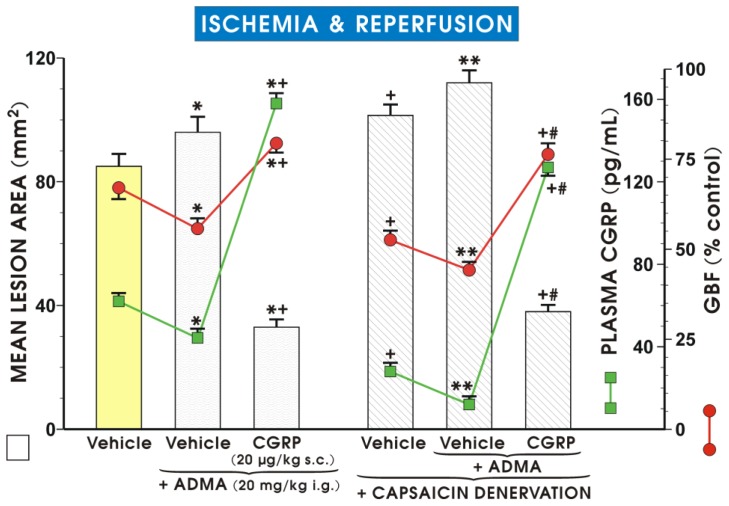
Mean lesion area of ischemia/reperfusion-induced damage, the alterations in the gastric blood flow (GBF), and the plasma CGRP levels in rats pretreated with ADMA alone or ADMA given in combination with CGRP in rats with or without capsaicin-induced denervation. Results are mean ± S.E.M of six to eight rats per group. Significant change (*p* < 0.05) as compared with the respective values in vehicle (saline)-control group is indicated by asterisk. A cross and asterisk indicate significant change (*p* < 0.05) as compared with vehicle-control group and with rats treated with vehicle or ADMA alone (left panel). Significant difference (*p* < 0.05) between the respective values in the vehicle (saline)-control group and the vehicle-control with capsaicin denervation, is indicated by a cross. A double asterisk denotes the significant change (*p* < 0.05) as compared with the respective values in vehicle-controls treated with ADMA without capsaicin denervation. A cross and a hash indicate significant change (*p* < 0.05) compared to the values obtained in capsaicin-denervated rats treated with ADMA without concurrent treatment with CGRP.

Figure 4 shows that the plasma CGRP level is significantly lower in rats pretreated with ADMA compared to the vehicle-control pretreated rats exposed to I/R (*p* < 0.05). Capsaicin-induced ablation of sensory nerves further reduced the plasma CGRP levels in the presence of ADMA. Moreover, ADMA administered to rats with capsaicin denervation markedly increased the mean lesion area and significantly decreased GBF compared to the ADMA-treated control group without this denervation (Figure 4). Concurrent treatment with CGRP reversed the increase in the mean lesion area, the fall in plasma CGRP level, and GBF induced by ADMA in capsaicin-denervated animals (*p* < 0.05) (Figure 4).

**Figure 5.**
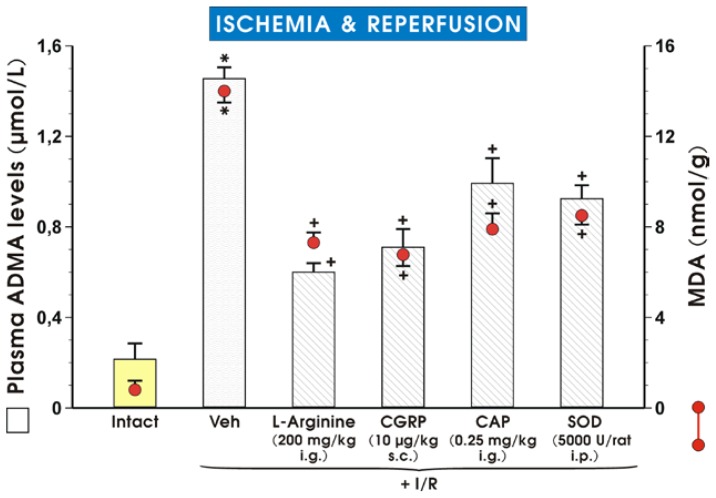
Plasma ADMA levels and MDA concentration in the gastric mucosa of intact rats, and those pretreated with vehicle (saline), l-arginine (200 mg/kg i.g.), CGRP (10 μg/kg s.c.), capsaicin (CAP) (applied in small protective dose (0.25 mg/kg i.g.)) or superoxide dismutase (SOD, 5000 U/rat i.p.) and exposed to 3.5 h of ischemia/reperfusion. Results are mean ± S.E.M of 7 rats per group. Significant change (*p* < 0.05) as compared with the intact group, is indicated by an asterisk. A cross indicates significant change (*p* < 0.05) as compared with vehicle-pretreated rats exposed to 3.5 h of I/R.

Results presented in Figure 5 confirm our previous observation [8] that plasma ADMA levels and malondialdehyde (MDA) concentration in gastric mucosa are significantly increased in rats exposed to I/R (*p* < 0.05) as compared with intact rats that did not undergo any procedures. The increase of plasma level of ADMA following the gastric I/R injury demonstrated in our previous work [8] was used in this present study as reference value to compare values of plasma ADMA levels in rats pretreated with a small “protective” dose of capsaicin [18] and CGRP, both affording significant gastric protection against I/R injury. As shown in Figure 5, the plasma ADMA levels and MDA content were significantly elevated in rats exposed to I/R. Pretreatment with l-arg, CGRP, capsaicin or superoxide dismutase (SOD), a potent antioxidant, significantly decreased plasma ADMA levels and MDA concentration in gastric mucosa (*p* < 0.05).

**Figure 6.**
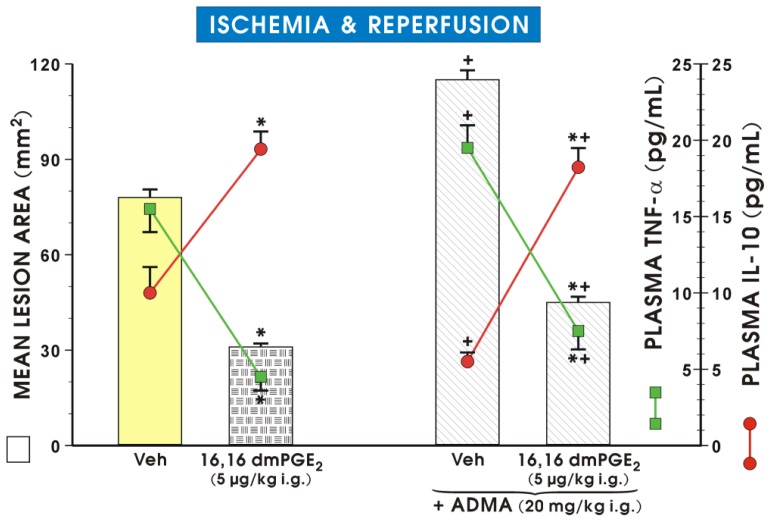
Mean lesion area, the alterations in GBF, and the plasma concentrations of proinflammatory cytokine TNF-α and anti-inflammatory cytokine IL-10 in rats pretreated with ADMA alone or ADMA applied in combination with synthetic prostaglandin E_2_ derivative, 16,16 dmPGE_2_, and exposed 30 min later to gastric ischemia/reperfusion. Results are mean ± S.E.M of 8 rats per group. Significant change (*p* < 0.05) as compared with the respective values in the vehicle (saline)-control group, is indicated by an asterisk. A cross indicates the significant difference (*p* < 0.05) between the values obtained in vehicle-controls with and without ADMA administration. An asterisk and a cross indicate significant change (*p* < 0.05) as compared with the vehicle group in the presence of ADMA.

Pretreatment with ADMA significantly increased the plasma level of pro-inflammatory cytokine TNF-α and produced a significant decrease in the plasma level of anti-inflammatory cytokine IL-10 as compared to the vehicle-control group (Figure 6). In rats pretreated with 16,16 dimethyl (dm) PGE_2_, a synthetic prototype of a cytoprotective agent [29], a significant decrease in plasma TNF-α level was observed, and the area of I/R-induced gastric lesions was significantly attenuated by pretreatment with this arachidonate metabolite analog. The pretreatment with dmPGE_2_ also reversed the decrease in plasma IL-10 levels and the fall in the GBF observed in rats exposed to I/R with or without the concurrent treatment with ADMA (Figure 6).

According to the data presented, ADMA could be a noxious factor, which enhances the acute mucosal damage within the gastrointestinal (GI) tract. First, we confirmed that plasma ADMA concentration was enhanced in rats with I/R injury [8] along with a profound rise in the gastric MDA content in these animals. Second, the evidence presented herein indicates that pretreatment with ADMA markedly decreased GBF and the luminal NO concentration, which resulted in an enhancement of the I/R-induced lesions. In another study, Li *et al.* [27] demonstrated that treatment with exogenous ADMA aggravated the ethanol-induced gastric lesions because ADMA inhibited NO release. In addition, Wang *et al.* [26] observed that ADMA content in gastric juice was markedly elevated in rats with ethanol-, indomethacin-, and cold stress-induced gastric lesions. Our results are in keeping with these observations and demonstrate that this asymmetric arginine derivative inhibits NO synthase activity and NO content while exacerbating I/R-induced gastric damage, as it was originally observed in the animal model of myocardial reperfusion injury [31].

In previous studies [32,33], the inhibition of NO synthesis by *N*(*G*)-nitro-l-arginine methyl ester (l-NAME) prior to ischemia aggravated I/R-induced gastric damage, but administration of this inhibitor prior to reperfusion reduced the I/R damage. We demonstrated in this study that ADMA aggravated I/R-induced gastric damage and l-arg, the substrate for NO synthesis, partially attenuated the aggravation of I/R injury induced by ADMA which is consistent with the two original observations by Kim *et al.* [32] and Kobata *et al.* [33] concerning similar aggravation effects of synthetic NO synthase inhibitor, l-NAME on I/R-induced gastric mucosal damage. Indeed, ADMA is considered a novel cardiovascular risk factor and its deleterious effect on endothelial NO-synthase activity in the cardiovascular system may be antagonized by concomitant supplementation with l-arg [34]. Our results are corroborative with this notion and demonstrate that pretreatment with exogenous l-arg, which afforded gastroprotection and hyperemia in the stomach, also decreased elevated plasma levels of ADMA and decreased MDA concentrations in the gastric mucosa in rats exposed to I/R. Taken together, these results confirm the important action of ADMA and l-arg in the severity of mucosal and cardiac damage and protection of the GI-tract and cardiovascular systems.

Our present study revealed that pretreatment with ADMA, which dose-dependently raised plasma ADMA increments, and also dose-dependently reduced the gastric luminal NO content. Of note, the inhibitory effect of ADMA on luminal NO release was documented both after completion of ischemia just before reperfusion, and was also observed at the end of I/R. Previous studies documented that NO could be essential for the mechanism of gastroprotection, in part, by NO-inhibition of gastric acid secretion [35]. NO inhibits gastric mucosal acid secretory activity via suppression of gastrin-induced histamine release through a pathway in which NO activates guanylate cyclase [36]. This action has been documented by a NO-induced increase in cGMP levels and a reduction of gastrin-induced calcium influx [36]. Furthermore, NO, as a new type of gastric acid inhibitor is expected to decrease histamine levels in the stomach [37]. Since hyperacidity is considered to be one of the risk factors responsible for impairment of the gastric mucosal barrier that may contribute to peptic ulcer disease, the ADMA-induced inhibition of NO release in our study can explain the enhanced susceptibility of gastric mucosa to lesions induced by I/R where gastric acid plays a role [38]. The gastric mucosal and luminal environment act as a significant source of nitrating and nitrosating agents, because at acidic pH levels in the stomach, the nitrite generates different nitrogen oxides depending on the local microenvironment (redox status, gastric content, pH, inflammatory conditions), including NO, nitrogen dioxide (NO_2_), dinitrogen trioxide (N_2_O_3_), and peroxynitrite [39]. Thus, both the gastric lumen and mucosa contain putative targets for nitration in the form of endogenous and exogenous proteins exerting an impact on local processes including ulcerogenesis.

Our data indicates that ADMA may attenuate the protective activity of a potent vasodilator, CGRP, which is released from afferent sensory nerve endings in the rat stomach. This suggests that ADMA can interact with the activity of sensory afferent nerves. We demonstrated previously that the activation of sensory afferent nerves helps to maintain a physiological gastric mucosal barrier against the damaging activity of various factors [40,41]. In our present study, pretreatment with both CGRP or capsaicin applied at the small dose that was shown to afford gastroprotection against acute gastric injury [18], decreased plasma concentration of ADMA and the lipid peroxidation product MDA. Moreover, pretreatment with ADMA further exacerbated the damage in rats with functional ablation of sensory nerves caused by capsaicin in gastric mucosa exposed to I/R. This exacerbating effect of ADMA in capsaicin-denervated rats was accompanied by a marked fall in GBF and a further decrease in plasma CGRP concentration compared to those observed in rats with capsaicin denervation alone. These effects were reversed by the supplementation of CGRP co-administered with ADMA in rats with capsaicin denervation. This reveals a new mechanism of ADMA-induced exacerbation of I/R injury by an impairment of vasodilatory neuropeptides such as CGRP, released from these nerves. Taken together, CGRP released from afferent sensory nerve endings is influenced by ADMA because its release is decreased by treatment with this NO synthesis inhibitor along with the aggravation of gastric mucosal I/R injury.

Herein, we have demonstrated an increased plasma level of proinflammatory cytokine TNF-α and decreased anti-inflammatory IL-10 plasma level associated with ADMA administration in animals exposed to I/R. This is corroborative with previously observed elevations of TNF-α and IL-1β plasma levels in animals subjected to gastric erosions induced by water immersion and restraint stress [13]. This increase in proinflammatory cytokines in damaged gastric mucosa could be due to inflammasomes, which are the multi-protein complexes that are activated by pathogen and danger associated molecular pattern molecules (PAMP and DAMPs, respectively), leading to the processing of IL-1β and IL-18 by caspase-1 [42]. Involvement of the nucleotide-binding oligomerisation domain receptors protein (NLRP) inflammasomes have recently been reported in gastrointestinal immune responses [43], myocardial I/R injury [44], and acute stroke [45]. Thus, the importance of IL-1β which functions as a prominent and early mediator for inflammation in the I/R injury cannot be excluded as a primary mediator of ADMA-induced exacerbation of gastric I/R lesions. But this hypothesis requires further study. Among the factors capable of exerting an inhibitory effect on ADMA-induced exaggeration of I/R lesions were PGs, which are considered to be prototypes of gastric cytoprotection *in vivo* [46] and *in vitro* [47]. We have previously demonstrated that pretreatment with exogenous 16,16 dmPGE_2_ exerts gastroprotection against I/R-induced gastric damage [40], and PG were also reported to act as primary mediators of gastric ischemic preconditioning known to exhibit a potent protective activity against I/R-induced gastric lesions [48]. Indeed, as shown in this study, pretreatment with the PGE_2_ analogue attenuated gastric I/R injury in the presence of ADMA, abrogated the increase in plasma level of TNF-α, and counteracted the apparent fall in anti-inflammatory cytokine IL-10 observed in I/R animals treated with this NO synthase inhibitor. This indicates that exogenous PG could be considered effective agents in the protection against ADMA perpetuation of the lesions induced by I/R.

Moreover, we demonstrated that the application of antioxidative SOD decreased plasma levels of ADMA and gastric tissue concentrations of MDA, which were both increased after exposure to I/R. This corresponds to our previous observation that treatment with ADMA resulted in decreased mRNA expression for SOD [13]. Taken together, ADMA seems to enhance the lipid peroxidation product MDA by inhibition of SOD, and this may contribute to exacerbation of gastric damage caused by I/R.

Our results are in agreement with the observation by Wu *et al.* [49] that the increased lung damage induced by cerebral I/R injury in rats is correlated with elevated plasma ADMA levels. Moreover, we also observed elevated plasma ADMA concentrations of rats exposed to stress [8]. Liu *et al.* [50] demonstrated that stimulation of sensory nerves resulting in CGRP release caused a decrease in ADMA concentration and ameliorated the damaging effect of ethanol on gastric mucosa. Our data refers to Chen *et al.* [51] who observed that cultured dorsal root ganglia neural cells co-incubated with ADMA decreased CGRP mRNA expression and inhibited the release of CGRP in the culture medium. However, Lu *et al.* [52] observed that the ADMA level in serum is increased more in aged (24 months) rats than in young animals (six months), but in their study no difference in mRNA expression for CGRP in ADMA-treated rats was noted. In contrast, CGRP production was decreased in spontaneously hypertensive (SHR) rats and this effect was accompanied by elevated plasma levels of ADMA [53]. In our present study, the supplementation of ADMA-treated rats with exogenous CGRP attenuated I/R-induced gastric lesions potentiated by ADMA and reversed in part, the accompanying decrease in GBF and plasma CGRP levels in rats with or without capsaicin denervation in the presence of ADMA.

## 3. Experimental Section

### 3.1. Animals, Ischemia/Reperfusion Injury, Afferent Sensory Nerves Ablation

Male Wistar rats with average weight of about 250 g were fasted for 24 h before the exposure to I/R with free access to drinking water. The study was approved by the Institutional Animal Care and Use Committee of Jagiellonian University Medical College in Cracow, Poland and run in accordance with the statements of the Helsinki Declaration regarding handling of experimental animals.

Acute I/R gastric lesions were induced in 120 rats as described previously [38,54,55]. Briefly, under pentobarbital anesthesia (60 mg/kg i.p.), the abdomen was opened, and the celiac artery was identified and clamped for 30 min followed by removal of the clamp to obtain reperfusion. After 3 h of I/R the gastric lesions were scored and the functional parameters described below were determined.

Rats with intact sensory nerves (series A) were randomized to the groups (6–8 rats each) and were administered 30 min before I/R with: (1) ADMA (0.1–40 mg/kg i.g.); (2) l-arg (200 mg/kg i.g.); (3) capsaicin (0.25 mg/kg i.g.); and (4) CGRP (10 μg/kg s.c.) alone or applied in combination with ADMA administered in the dose of 20 mg/kg i.g., which was selected based on the dose-dependent enhancement of the area of I/R injury in rats treated with this NO synthase inhibitor. In addition, the effect of the FXR agonist, GW4064 (3 mg/kg i.p.), which has been shown to increase the expression of DDAH-1 and to decrease the circulating levels of ADMA [15–17], was measured in rats of series A with and without ADMA administration, and exposed to 3.5 h of I/R.

A separate group of rats (series B) was administered capsaicin for three days at a dose of 25, 50 and 50 mg/kg (total dose: 125 mg/kg s.c.) to induce functional ablation of afferent sensory nerves as described previously [54]. After two weeks, rats of series B were pretreated with ADMA (20 mg/kg i.g.) alone or in combination with CGRP (10 μg/kg s.c.) and exposed to standard 3.5 h of I/R. In all series of rats, vehicle-(saline) control groups, exposed to I/R were used as a comparison for particular compounds tested in this experimental model of gastric lesions.

Rats of series C were pretreated with l-arg (200 mg/kg i.g.), CGRP (10 μg/kg i.g.), capsaicin (0.25 mg/kg i.g.) and SOD (5000 U/rat i.p.), 30 min before the exposure to standard I/R. Rats of series D received the pretreatment with vehicle (saline) or 16, 16 dm PGE_2_ (5 μg/kg i.g.) with or without the combination of ADMA (20 mg/kg i.g.) applied 30 min before the I/R.

In series E, rats were administered saline and exposed to 30 min of ischemia alone, and blood samples were collected for NO luminal content and ADMA plasma level determination.

All tested compounds were of analytical grade and were purchased from Sigma-Aldrich (Schelldorf, Germany).

### 3.2. Determination of Gastric Blood Flow and the Area of Gastric Lesions

After the end of 3 h of I/R, the animals of series A–D were anesthetized with pentobarbital (60 mg/kg i.p.), the abdomen was opened, and the stomach was exposed to measure GBF by means of the H_2_-gas clearance technique as described previously [54,56]. The GBF was measured in the fundic part of the gastric mucosa not involving mucosal lesions. Average values of three measurements were determined and expressed as a percentage of the change of the value determined in intact rat stomachs. The area of gastric lesions was determined with computer planimetry (Morphomat, Carl Zeiss, Berlin, Germany) by a person who did not know which experimental group the animals belonged to.

### 3.3. Determination of Luminal NO Release and the Gastric Mucosal MDA Content

The lumen concentration of NO was quantified indirectly as nitrate (NO_3_^−^) and nitrite (NO_2_^−^) levels in the gastric contents, using the nitrate/nitrite kit purchased from Cayman Lab, Ann Arbor, MI, USA as described previously [57]. This method is based on the Griess reaction and generation of chromophore absorbing at 595 nm, according to the original procedure [58]. Since NO released by epithelial cells into the gastric lumen is quickly transformed into NO_3_^−^ and NO_2_^−^ [59], we photometrically measured the sum of both of these products of NO-synthase as an index of NO production by the enzyme in the gastric mucosa. For this purpose, the gastric contents were aspirated at the end of 3.5 h of I/R just before removal of the stomach following the i.g. injection of 1 mL of saline to wash out the lumen contents. In some experiments, the gastric luminal content was collected immediately after the end of 30 min of ischemia in order to check whether treatment with ADMA results in early inhibition of NO before the subsequent period of reperfusion.

After centrifugation for 10 min at 3000 rpm, the samples were mixed with Griess reagent (Cayman Lab, Ann Arbor, MI, USA).

To determine MDA tissue concentration the colorimetric assay for lipid peroxidation (Bioxytech LPO-586, Oxis, Portland, OR, USA) was used as described previously [13]. In brief, about 600 mg of gastric mucosa was excised, quickly washed in a test tube and 20 μL 0.5 M butylated hydroxytoluene was added in order to prevent sample oxidation. This sample was subsequently homogenized in 20 mM Tris for 15 s. in pH 7.4. Then homogenate was centrifuged (3000× *g* at 4 °C for 10 min) and the clear supernatant obtained was immediately stored at −80 °C prior to testing. The colorimetric assay used to determine MDA concentration in gastric mucosa is based on the reaction of a chromogenic reagent *N*-methyl-2-phenylindole with MDA and 4-hydroksynonenal (4-HNE) at 45 °C which yields a stable chromophore with maximal absorbance at 586 nm, then is subsequently analyzed using a micro plate reader.

### 3.4. Assessment of Plasma Levels of ADMA, CGRP and Proinflammatory and Anti-Inflammatory Cytokines

At the end of 3.5 h of I/R and immediately after GBF measurement was completed, the blood samples (about 3 mL) were taken from the *vena cava* for the measurement of plasma ADMA, CGRP, proinflammatory (TNF-α) and anti-inflammatory (IL-10) cytokines as described previously [13,60,61].

The CGRP level was measured by radioimmunoassay (RIA) in blood samples taken from the *vena cava*, using a specific CGRP antibody as described previously [60,61]. The rat CGRP-RIA kit was purchased from Phoenix Pharmaceuticals, Inc. (Belmont, CA, USA). The assay specificity showed 100% cross-reactivity with rat CGRP and 35.5% cross-reactivity with human CGRP and no cross-reactivity with rat amylin, calcitonin and somatostatin-14. The limit of assay sensitivity was 32 pg per tube; the intra-assay variation was less than 9% and the inter-assay variation was less than 4%.

The concentration of ADMA in the blood was determined using ADMA direct rat ELISA kit manufactured by Immunodiagnostik AG (Enzo Life Sciences GMBH, Lorrach, Germany) according to the procedure recommended by the manufacturer and described previously [13]. Briefly, the aliquots (50 μL) of the pretreated standards, controls and samples were pipetted into wells of the microtitre plate and the antiserum solution (ADMA antibody, 50 μL) was added to each well. The plate was incubated for 15 h at 2–8 °C and washed four times with wash buffer. Subsequently, the enzyme conjugate solution (100 μL) was added to each well and then the microtitre plate was incubated for one hour at room temperature. The wells were again washed four times and the substrate solution (100 μL) was pipetted into the wells and the plate was incubated for 25 min. Then the reaction was stopped with stopping solution and the optical density was read at 450 nm using a microtitre plate reader. The limit of assay sensitivity was 3 pg per tube; the intra-assay variation was less than 7% and the inter-assay variation less than 4%.

The plasma TNF-α and IL-10 concentrations levels were determined using a solid phase sandwich ELISA (BioSource International Inc., Camarillo, CA, USA) according to the manufacturer’s instructions. Briefly, each sample (50 μL) was incubated with biotinylated antibodies specific for rat TNF-α a nd I L-10 washed three times with assay buffer and finally conjugated with streptavidin peroxidase to form a complex with a stabilized chromogen as described before [13].

### 3.5. Statistical Analysis

Results of the experiment were expressed as mean ± S.E.M and the statistical analysis was performed with ANOVA test and Tukey *post hoc* test where appropriate. Differences between estimates of effects were considered significant at *p* < 0.05.

## 4. Conclusions

We demonstrated that ADMA can exacerbate gastric injury when gastric mucosa is compromised by I/R. The deleterious effect of ADMA administration on I/R gastric lesions was ameliorated by the FXR agonist, GW4064. This beneficial protective effect of GW4064 could be explained in part, by an increase in DDAH1, which subsequently increased the metabolism of ADMA, and increased NO bioavailability. Moreover, we confirmed that the plasma circulating ADMA level and gastric MDA content are increased in animals exposed to I/R. We found that these effects are attenuated by l-arginine, SOD, and capsaicin, the latter applied in the small dose known to stimulate sensory afferent nerves and resulting in the release of vasodilatory mediators such as CGRP [18]. ADMA-induced aggravation of I/R gastric damaged mucosa was accompanied by a decrease in GBF and a rise in plasma ADMA levels, which correlated with reduced NO release into the gastric lumen and was accompanied by the impairment of CGRP release from sensory nerves. The ADMA aggravation of I/R damage in rats with intact sensory nerves and the accompanying decrease in plasma CGRP concentration in capsaicin-denervated rats was counteracted by supplementation of exogenous CGRP added to ADMA-treated rats with and without capsaicin denervation. We conclude that the impairment of gastric mucosal defense by treatment with ADMA, as manifested by exacerbation of I/R-induced gastric lesions, involves a decrease in gastric microcirculation mediated by the fall in NO and CGRP, and the enhanced lipid peroxidation and proinflammatory action of this NO synthase inhibitor.

## Figures and Tables

**Table 1.** Effect of pretreatment with vehicle (saline) or ADMA administered i.g. in graded doses ranging from 0.1 up to 40 mg/kg i.g. on the plasma levels of ADMA in rats exposed to 3.5 h of ischemia/reperfusion. Mean ± S.E.M of 6 rats per each group. An asterisk indicates a significant (*p* < 0.05) increase as compared with vehicle-control. An asterisk and cross indicate a significant difference (*p* < 0.05) when compared with the vehicle and ADMA applied in doses 1 or 10 mg/kg.

Experimental group	Plasma ADMA concentration (μmol/L)
Vehicle	1.46 ± 0.22
ADMA (0.1 mg/kg i.g.)	1.83 ± 0.36
ADMA (1 mg/kg i.g.)	2.37 ± 0.46 *
ADMA (10 mg/kg i.g.)	2.65 ± 0.34 *
ADMA (20 mg/kg i.g.)	3.62 ± 0.43 *+
ADMA (40 mg/kg i.g.)	4.95 ± 0.58 *+

**Table 2.** Effect of pretreatment with vehicle (saline) or ADMA (20 mg/kg i.g.) on the luminal NO concentration measured in rats after ischemia alone (30 min), and in those with ischemia (30 min) followed by reperfusion (3 h). Mean ± S.E.M of 6 rats per each group. An asterisk indicates a significant decrease (*p* < 0.05) as compared to the values obtained in vehicle-control rats after ischemia alone, or after ischemia/reperfusion.

Experimental group	Luminal NO (μmol/L)
After ischemia before reperfusion	

Vehicle	15.2 ± 2.06
ADMA (20 mg/kg i.g.)	7.6 ± 0.08 *

After ischemia/reperfusion	

Vehicle	14.6 ± 1.35
ADMA (20 mg/kg i.g.)	6.3 ± 0.04 *

## References

[1] Zakrzewicz D., Eickelberg O. (2009). From arginine methylation to ADMA: A novel mechanism with therapeutic potential in chronic lung diseases. BCM Pulm. Med..

[2] Leiper J., Vallance P. (1999). Biological significance of endogenous methylarginines that inhibit nitric oxide synthases. Cardiovasc Res..

[3] Leiper J., Vallance P. (2006). The synthesis and metabolism of asymmetric dimethylarginine (ADMA). Eur. J. Clin. Pharmacol..

[4] Beltowski J., Kedra A. (2006). Asymmetric dimethylarginine (ADMA) as a target for pharmacotherapy. Pharmacol. Rep..

[5] Wilcken D.E., Sim A.S., Wang J., Wang X.L. (2007). Asymmetric dimethylarginine (ADMA) in vascular renal and hepatic disease and the regulatory role of l-arginine on its metabolism. Mol. Genet. Metab..

[6] Vallance P., Leiper J. (2004). Cardiovascular biology of the asymmetric dimethylarginine dimethylaminohydrolase pathway. Arterioscler. Thromb. Vasc. Biol..

[7] Cardounel A.J., Cui H., Samouilov A., Johnson W., Kearns P., Tsai A.L., Berka V., Zweier J.L. (2007). Evidence for the pathophysiological role of endogenous methylarginines in regulation of endothelial NO production and vascular function. J. Biol. Chem..

[8] Szlachcic A., Krzysiek-Maczka G., Pajdo R., Targosz A., Magierowski M., Jasnos K., Drozdowicz D., Kwiecien S., Brzozowski T. (2013). The impact of asymmetric dimethylarginine (ADMA) the endogenous nitric oxide (NO) synthase inhibitor to the pathogenesis of gastric mucosal damage. Curr. Pharm. Des..

[9] Zhang G.G., Bai Y.P., Chen M.F., Shi R.Z., Jiang D.J., Fu Q.M., Tan G.S., Li Y.J. (2008). Asymmetric dimethylarginine induces TNF-alpha production via ROS/NF-kappaB dependent pathway in human monocytic cells and the inhibitory effect of retinioside C. Vasc. Pharmacol..

[10] Chen M., Li Y., Yang T., Wang Y., Bai Y., Xie X. (2008). ADMA induces monocyte adhesion via activation of chemokine receptors in cultured THP-1 cells. Cytokine.

[11] Landim M.B., Casella Filho A., Chagas A.C. (2009). Asymmetric dimethylarginine (ADMA) and endothelial dysfunction: implications for atherogenesis. Clinics.

[12] Zhang Z., Zhou Y., Zou Y.Y., Wang L., Yang Z.C., Guo R., Li D., Peng J., Li Y.J. (2008). Detrimental effects of nicotine on the acute gastric mucosal injury induced by ethanol: Role of asymmetric dimethylarginine. Can. J. Physiol. Pharmacol..

[13] Kwiecien S., Ptak-Belowska A., Krzysiek-Maczka G., Targosz A., Jasnos K., Magierowski M., Szczyrk U., Brzozowski B., Konturek S.J., Konturek P.C. (2012). Asymmetric dimethylarginine an endogenous inhibitor of nitric oxide synthase interacts with gastric oxidative metabolism and enhances stress-induced gastric lesions. J. Physiol. Pharmacol..

[14] Ogawa T., Kimoto M., Sasaoka K. (1987). Occurrence of a new enzyme catalyzing the direct conversion of NG NG-dimethyl-l-arginine to l-citrulline in rats. Biochem. Biophys. Res. Commun..

[15] Liu X., Fassett J., Wei Y., Chen Y. (2013). Regulation of DDAH1 as a potential therapeutic target for treating cardiovascular diseases. Evid. Based Complement. Alternat. Med..

[16] Vaquero J., Briz O., Herraez E., Muntané J., Marin J.J. (2013). Activation of the nuclear receptor FXR enhances hepatocyte chemoprotection and liver tumor chemoresistance against genotoxic compounds. Biochim. Biophys. Acta.

[17] Li J., Wilson A., Gao X., Kuruba R., Liu Y., Poloyac S., Pitt B., Xie W., Li S. (2009). Coordinated regulation of dimethylarginine dimethylaminohydrolase-1 and cationic amino acid transporter-1 by farnesoid X receptor in mouse liver and kidney and its implication in the control of blood levels of asymmetric dimethylarginine. J. Pharmacol. Exp. Ther.

[18] Brzozowski T., Konturek S.J., Sliwowski Z., Pytko-Polonczyk J., Szlachcic A., Drozdowicz D. (1996). Role of capsaicin-sensitive sensory nerves in gastroprotection against acid-independent and acid-dependent ulcerogens. Digestion.

[19] Villegas I., Martin A.R., Toma W., de la Lastra C.A. (2004). Rosiglitazone an agonist of peroxisome proliferator-activated receptor gamma protects against gastric ischemia-reperfusion damage in rats: Role of oxygen free radicals generation. Eur. J. Pharmacol..

[20] Flögel U., Decking U.K., Gödecke A., Schrader J. (1999). Contribution of NO to ischemia-reperfusion injury in the saline-perfused heart: A study in endothelial NO synthase knockout mice. J. Mol. Cell. Cardiol..

[21] Pacher P., Beckman J.S., Liaudet L. (2007). Nitric oxide and peroxynitrite in health and disease. Physiol. Rev..

[22] Chatterjee P.K., Patel N.S., Kvale E.O., Cuzzocrea S., Brown P.A., Stewart K.N., Mota-Filipe H., Thiemmermann C. (2002). Inhibition of inducible nitric oxide synthase reduces renal ischemia/reperfusion injury. Kidney Int..

[23] Andelová E., Barteková M., Pancza D., Styk J., Ravingerová T. (2005). The role of NO in ischemia/reperfusion injury in isolated rat heart. Gen. Physiol. Biophys..

[24] Phan L.H., Hickey M.J., Niazi Z.B., Stewart A.G. (1994). Nitric oxide synthase inhibitor nitro-iminoethyl-l-ornithine reduces ischemia-reperfusion injury in rabbit skeletal muscle. Microsurgery.

[25] Andrews F.J., Malcontenti-Wilson C., O’Brien P.E. (1994). Protection against gastric ischemiareperfusion injury by nitric oxide generators. Dig. Dis. Sci..

[26] Wang L., Zhou Y., Peng J., Zhang Z., Jiang D.J., Li Y.J. (2008). Role of endogenous nitric oxide synthase inhibitor in gastric mucosal injury. Can. J. Physiol. Pharmacol..

[27] Li L., Luo X.J., Liu Y.Z., Zhang Y.S., Yuan Q., Tan N., Xiang D.X., Peng J. (2010). The role of the DDAH-ADMA pathway in the protective effect of resveratrol analog BTM-0512 on gastric mucosal injury. Can. J. Physiol. Pharmacol..

[28] Zhang Z., Zou Y.Y., Li F.J., Hu C.P. (2011). Asymmetric dimethylarginine: A novel biomarker of gastric mucosal injury?. World J. Gastroenterol..

[29] Robert A. (1983). Cytoprotection of the gastrointestinal mucosa. Adv. Intern. Med.

[30] Brzozowski T., Konturek P.C., Konturek S.J., Brzozowska I., Pawlik T. (2005). Role of prostaglandins in gastroprotection and gastric adaptation. J Physiol Pharmacol.

[31] Stühlinger M.C., Conci E., Haubner B.J., Stocker E.M., Schwaighofer J., Cooke J.P., Tsao P.S., Pachinger O., Metzler B. (2007). Asymmetric dimethyl l-arginine (ADMA) is a critical regulator of myocardial reperfusion injury. Cardiovasc. Res..

[32] Kim H., Hwan Kim K. (2001). Role of nitric oxide and mucus in ischemia/reperfusion-induced gastric mucosal injury in rats. Pharmacology.

[33] Kobata A., Kotani T., Komatsu Y., Amagase K., Kato S., Takeuchi K. (2007). Dual action of nitric oxide in the pathogenesis of ischemia/reperfusion-induced mucosal injury in mouse stomach. Digestion.

[34] Boger R.H. (2004). Asymmetric dimethylarginine an endogenous inhibitor of nitric oxide synthase explains the “l-arginine paradox” and acts as a novel cardiovascular risk factor. J Nutr.

[35] Berg A., Redéen S., Grenegård M., Ericson A.C., Sjöstrand S.E. (2005). Nitric oxide inhibits gastric acid secretion by increasing intraparietal cell levels of cGMP in isolated human gastric glands. Am. J. Physiol. Gastrointest. Liver Physiol.

[36] Combet E., Preston T., McColl K.E. (2010). Development of an *in vitro* system combining aqueous and lipid phases as a tool to understand gastric nitrosation. Rapid Commun. Mass Spectrom.

[37] Shibata N., Matsui H., Yokota T., Matsuura B., Maeyama K., Onji M. (2006). Direct effects of nitric oxide on histamine release from rat enterochromaffin-like cells. Eur. J. Pharmacol.

[38] Brzozowski T., Konturek P.C., Konturek S.J., Drozdowicz D., Kwiecien S., Pajdo R., Bielanski W., Hahn E.G. (2000). Role of gastric acid secretion in progression of acute gastric erosions induced by ischemia-reperfusion into gastric ulcers. Eur. J. Pharmacol..

[39] Rocha B.S., Gago B., Barbosa R.M., Lundberg J.O., Radi R., Laranjinha J. (2012). Intragastric nitration by dietary nitrite: implications for modulation of protein and lipid signaling. Free Radic. Biol. Med.

[40] Brzozowski T., Konturek P.C., Konturek S.J., Pajdo R., Kwiecien S., Pawlik M., Drozdowicz D., Sliwowski Z., Pawlik W.W. (2004). Ischemic preconditioning of remote organs attenuates gastric ischemia-reperfusion injury through involvement of prostaglandins and sensory nerves. Eur. J. Pharmacol..

[41] Kwiecien S., Pawlik M.W., Sliwowski Z., Kwiecien N., Brzozowski T., Pawlik W.W., Konturek S.J. (2007). Involvement of sensory afferent fibers and lipid peroxidation in the pathogenesis of stress-induced gastric mucosa damage. J. Physiol. Pharmacol..

[42] Mogensen T.H. (2009). Pathogen recognition and inflammatory signaling in innate immune defenses. Clin. Microbiol. Rev.

[43] Elinav E., Henao-Mejia J., Flavell R.A. (2013). Integrative inflammasome activity in the regulation of intestinal mucosal immune responses. Mucosal Immunol.

[44] Sandanger ⊘, Ranheim T., Vinge L.E., Bliksøen M., Alfsnes K., Finsen A.V., Dahl C.P., Askevold E.T., Florholmen G., Christensen G. (2013). The NLRP3 inflammasome is up-regulated in cardiac fibroblasts and mediates myocardial ischemia-reperfusion injury. Cardiovasc. Res.

[45] Fann D.Y., Lee S.Y., Manzanero S., Chunduri P., Sobey C.G., Arumugam T.V. (2013). Pathogenesis of acute stroke and the role of inflammasomes. Ageing Res. Rev.

[46] Tarnawski A., Brzozowski T., Sarfeh I.J., Krause W.J., Ulich T.R., Gergely H., Hollander D. (1988). Prostaglandin protection of human isolated gastric glands against indomethacin and ethanol injury Evidence for direct cellular action of prostaglandin. J. Clin. Investig.

[47] Brzozowski T., Tarnawski A., Hollander D., Sekhon S., Krause W.J., Gergely H. (2005). Comparison of prostaglandin and cimetidine in protection of isolated gastric glands against indomethacin injury. J. Physiol. Pharmacol.

[48] Pajdo R., Brzozowski T., Konturek P.C., Kwiecien S., Konturek S.J., Sliwowski Z., Pawlik M., Ptak A., Drozdowicz D., Hahn E.G. (2001). Ischemic preconditioning the most effective gastroprotective intervention: involvement of prostaglandins nitric oxide adenosine and sensory nerves. Eur. J. Pharmacol.

[49] Wu Y.H., Zhang X., Wang D.H. (2011). Role of asymmetric dimethylarginine in acute lung injury induced by cerebral ischemia/reperfusion injury in rats. Nan Fang Yi Ke Da Xue Xue Bao.

[50] Liu Y.Z., Zhou Y., Li D., Wang L., Hu G.Y., Peng J., Li Y.J. (2008). Reduction of asymmetric dimethylarginine in the protective effects of rutaecarpine on gastric mucosal injury. Can. J. Physiol. Pharmacol..

[51] Chen Q.Q., Li D., Guo R., Luo D., Yang J., Hu C.P., Li Y.J. (2008). Decrease in the synthesis and release of calcitonin gene-related peptide in dorsal root ganglia of spontaneously hypertensive rat: Role of nitric oxide synthase inhibitors. Eur J Pharmacol.

[52] Lu R., Zhu H.Q., Peng J., Li N.S., Li Y.J. (2002). Endothelium-dependent vasorelaxation and the expression of calcitonin gene-related peptide in aged rats. Neuropeptides.

[53] Li D., Chen B.M., Peng J., Zhang Y.S., Li X.H., Yuan Q., Hu C.P., Deng H.W., Li Y.J. (2009). Role of anandamide transporter in regulating calcitonin gene-related peptide production and blood pressure in hypertension. J Hypertens.

[54] Brzozowski T., Konturek P.C., Sliwowski Z., Pajdo R., Drozdowicz D., Kwiecien S., Burnat G., Konturek S.J., Pawlik W.W. (2006). Prostaglandin/cyclooxygenase pathway in ghrelin-induced gastroprotection against ischemia-reperfusion injury. J. Pharmacol. Exp. Ther..

[55] Wada K., Kamisaki Y., Kitano M., Kishimoto Y., Nakamoto K., Itoh T. (1996). A new gastric ulcer model induced by ischemia-reperfusion in the rat: Role of leukocytes on ulceration in rat stomach. Life Sci..

[56] Konturek S.J., Brzozowski T., Pytko-Polonczyk J., Drozdowicz D. (1995). Exogenous and endogenous cholecystokinin protects gastric mucosa against the damage caused by ethanol in rats Eur. J. Pharmacol..

[57] Konturek P.C., Brzozowski T., Sliwowski Z., Pajdo R., Stachura J., Hahn E.G., Konturek S.J. (1998). Involvement of nitric oxide and prostaglandins in gastroprotection induced by bacterial lipopolysaccharide. Scand. J. Gastroenterol..

[58] Green L.C., Tannenbaum S.R., Goldman P. (1981). Nitrate synthesis in the germ-free and conventional rat. Science.

[59] Ignarro L.J. (1993). Nitric oxide-mediated vasorelaxation. Tromb. Haemost..

[60] Brzozowski T., Konturek P.C., Sliwowski Z., Drozdowicz D., Burnat G., Pajdo R., Pawlik M., Bielanski W., Kato I., Kuwahara A. (2008). Gastroprotective action of orexin-A against stress-induced gastric damage is mediated by endogenous prostaglandins sensory afferent neuropeptides and nitric oxide. Regul. Pept..

[61] Peng J., Lu R., Deng H.W., Li Y.J. (2002). Involvement of alpha-calcitonin gene-related peptide in monophosphoryl lipid A-induced delayed preconditioning in rat hearts. Eur. J. Pharmacol..

